# Value-Added Foods: Composition, Sensory, and Consumer Research

**DOI:** 10.3390/foods13233952

**Published:** 2024-12-07

**Authors:** Witoon Prinyawiwatkul

**Affiliations:** School of Nutrition and Food Sciences, Louisiana State University, Agricultural Center, Baton Rouge, LA 70803, USA; wprinya@lsu.edu or wprinyawiwatkul@agcenter.lsu.edu

The term “value-added” continues to be a relevant topic for global research, especially when it is related to increased economic value, increased consumer satisfaction, food waste reduction, and sustainability. In general, adding value is a process of changing or transforming raw or pre-processed materials, agricultural commodities, or products from their original state to a more desirable state that satisfies consumer demands in terms of intrinsic and/or extrinsic qualities. “Value-added” is one of the production/marketing strategies driven by consumer needs and perceptions. Pertaining to the food industry, recycling generally refers to transforming wastes or by-products into a product or ingredient that can be further used to make something new and more valuable. Upcycling refers to the repurposing of materials (e.g., ugly produces) that would normally be discarded by grocery stores; hence, it leverages their values and creates a sustainable and resilient food system.

Consumer interest in safer, more sustainable, and healthier foods drives research on new value-added foods, new food ingredients, new food processing technologies, and novel packaging. These research foci, in turn, lead to positive changes in food quality including physical, chemical, and sensory properties, as well as health benefits. There must be a balance between the new ingredients, processing, and technologies to be implemented and their effects of sensory quality, food safety, availability, shelf life, consumer perception, affordability, and purchase decision of the final product.

In this Special Issue, let us begin with the concept of a zero-waste food system, which will benefit farmers, producers, consumers, and our planet. Within the global food supply chain, from farming, harvesting, pre-processing, processing, and food retailing to consumers, somewhere, somehow, by-products or waste materials are generated [1]. The fruit and vegetable industry contributes significantly to global annual post-harvest food waste, mainly due to product grading to meet consumer acceptability, and during processing, particularly for juice processing and pulp extraction. Residues of fruit and vegetable processing are valuable sources of bioactive compounds with high potential to be used as novel functional food ingredients [2,3] and potential applications in the treatment and prevention of human diseases. The growth of recycled and upcycled foods creates opportunities to provide value-added high-quality products to consumers while reducing food and agricultural waste, and has a positive impact on the environment and economy. Soontornwat et al. (2024) (Contribution 1) investigated how to effectively and economically upcycle the mangosteen pericarp into a value-added bioactive compound. To be specific, this research work was performed to obtain the effective recovery of α-mangostin from mangosteen pericarp, a byproduct collected from the mobile processing system. They reported that a “sequential slow freezing then hot air drying” process was the most operationally and economically viable scheme to process mangosteen pericarp. This processing scheme could recover the α-mangostin content at 78.9 mg/g of dry-weight of mangosteen pericarp. This scheme requires low initial capital investment and is easy to operate and maintain. In addition, since the potential users of this system are either small-scale farmers with limited financial resources or low-skilled workers, this scheme is, thus, an operationally and economically ideal option (Contribution 1).

Alternative proteins are fast growing and have gained popularity globally as consumers are looking for foods that are healthy, nutritious, and sustainable. Liu et al. (2024) (Contribution 2) published a comprehensive review summarizing scientific and technological aspects of six different types of proteins: dairy proteins, plant proteins, precision fermentation proteins, cell-cultured proteins, algal proteins, and mycoproteins. They also comprehensively analyzed and discussed some opportunities and challenges for each type of protein, which would prompt future research and development. They further emphasized that more research on upscaling, cost reduction, potential food applications, improved sensory properties, and consumer perception is critically needed (Contribution 2). Another alternative protein source is microalgae [4]. Microalgae have attracted considerable attention as an emerging food resource due to their fast growth rate and high nutritional value, including a variety of bioactive chemicals and unique lipids, proteins, essential amino acids, and carbohydrates [5]. Chlorella and Arthrospira platensis are at the forefront of the microalgal market due to their simple cultivation and high protein content and nutritional values. Çelekli et al. (2024) (Contribution 3) published a comprehensive review reporting commonly used microalgae in the food industry with regard to their advantages, opportunities, challenges, recent food applications, and new trends, particularly as functional food products.

Another new food source is edible insects which have been touted for complete protein, desirable fatty acid profiles, micronutrients, and bioactive compounds. Compared to traditional meat production, food products that are made of edible insects may provide a more sustainable and environmentally friendly protein source and contribute to a circular economy [6,7]. Despite the potential benefits of sustainable nutrition, Western consumers are reluctant to adopt entomophagy (consumption of insects). Gao et al. (2024) (Contribution 4) investigated the impact of different ways of information presentation on consumer perceptions of cricket-containing chocolate chip cookies (CCCs). They reported that different presenting formats (text, image(s), and/or combinations, as well as presenting an actual product) significantly influenced the willingness to consumer (WTC), acceptance, and purchase intent (PI) of CCCs. Specifically, showcasing the actual CCC product was effective in lessening the adverse attitudes toward edible insects, and emphasizing the health and environmental benefits to male consumers and early adopters could enhance the perceived acceptability of insect-based foods and, hence, increase their interest in food-containing insects. Developing new processing techniques to make insects more acceptable to consumers is a promising area of research. Through culinary innovation, insect-based food products can be promoted. If successfully integrated into the food system, edible insects could serve as sustainable and nutritious diets for future generations.

The editor hopes that the readers will find this Special Issue insightful, interesting, and useful for future research. The information presented in this SI should inspire and encourage future exploration of multidisciplinary research collaboration which would lead to more effective and efficient technologies for recycling and upcycling food and agricultural by-products and waste, and for producing and utilizing alternative new and sustainable food sources.
